# Tamsulosin plus Tadalafil compared with Tamsulosin alone for benign prostate hyperplasia in patients with or without erectile dysfunction: a meta-analysis and meta-regression of randomized controlled trials

**DOI:** 10.1007/s00345-025-05662-w

**Published:** 2025-05-11

**Authors:** Tallal Mushtaq Hashmi, Rohma Zia, Hadiah Ashraf, Momina Siddiqui, Ali Haider, Valencia Rumampouw, Momina Aslam Khan, Mushood Ahmed, Javed Iqbal

**Affiliations:** 1 Department of Medicine, Rawalpindi Medical University, Rawalpindi, Pakistan; 2 Newcastle University, Newcastle, UK; 3 Abbas Institute of Medical Sciences, Muzaffarabad, Pakistan; 4 Hamad Medical Corporation, P.O Box 3050, Doha, Qatar

**Keywords:** Benign prostatic hyperplasia, Erectile dysfunction, Tamsulosin, Tadalafil, Meta-analysis

## Abstract

**Background:**

Combination pharmacotherapy with tamsulosin plus tadalafil could be a superior strategy compared to conventional monotherapy with tamsulosin in patients with Benign prostatic obstruction (BPO) with or without erectile dysfunction.

**Methodology:**

A comprehensive search was conducted across PubMed, the Cochrane Library, and Embase to identify studies assessing the efficacy and safety of combination therapy compared with monotherapy in patients with BPO with or without erectile dysfunction. A random effects meta-analysis was performed with R version 4.4.1 using the 'meta' package.

**Results:**

We included eleven RCTs, with a combined total of 940 patients. Our analysis demonstrated that the combination therapy is associated with a greater reduction in overall IPSS (MD = − 2.78, 95% CI − 3.97 to − 1.59; P < 0.01), IIEF (MD = 2.98, 95% CI 1.64 to 4.33; P < 0.01), QoL score (MD = − 0.58, 95% CI − 0.86 to − 0.30; P < 0.01), PVR (MD = − 9.34, 95% CI = -15.52 to − 3.16; P < 0.01) and a significant improvement in Qmax (MD = 1.04, 95% CI 0.43 to 1.64; P < 0.01) as compared to tamsulosin alone. However, combination therapy resulted in a higher incidence of pain (OR = 5.66, 95% CI 2.56 to 12.52; P < 0.01) and other adverse events (OR = 2.97, 95% CI 1.60 to 5.49; P < 0.01).

**Conclusion:**

Combination therapy with tamsulosin and tadalafil demonstrated superior efficacy over tamsulosin monotherapy in reducing LUTS, improving quality of life, and enhancing erectile function. However, it was associated with a higher incidence of adverse effects.

**Supplementary Information:**

The online version contains supplementary material available at 10.1007/s00345-025-05662-w.

## Introduction

Benign prostatic obstruction (BPO) is histologically characterized by nonmalignant hyperplasia of both smooth muscles and epithelial tissue in the prostate. The condition leads to enlargement of the prostate, which can obstruct the urethra and cause a variety of lower urinary tract symptoms (LUTS) [1, 2]. BPO is common in aging men worldwide with significant negative impact on quality of life [3, 4]. For these patients the most common and straightforward treatment approach begins with lifestyle and behavior changes. If these adjustments are not enough, drug therapy is introduced [5]. Medications such as alpha blockers, PDE5 inhibitors, anticholinergics and beta-3 agonists are used either alone or in combinations to treat BPO/LUTS [6–9]. Tamsulosin is a targeted α1- adrenoceptor blocker that primarily affects the prostate, showing greater specificity for prostatic tissues compared to other α1-blockers. This selectivity allows it to relax the muscles in the prostate and bladder neck, improving urine flow in conditions like BPH while reducing the likelihood of side effects elsewhere in the body [10]. Tadalafil, a PDE5 inhibitor, is used to treat erectile dysfunction (ED), secondary to BPO by decreasing the levels of cyclic guanosine monophosphate (cGMP) in the penile tissue. It works by acting on the nitric oxide-cGMP pathway, which helps relax blood vessels and increase blood flow to the penis, making it easier to achieve an erection [11, 12]. Using tadalafil alone or with tamsulosin to treat both ED and lower urinary tract symptoms (LUTS) that are the most prominent effects of BPO, could lead to more personalized treatment plans. However, the main challenge is ensuring that the treatment is both effective and well-tolerated [13].

Our systematic review's objective is to assess and summarize the safety and effectiveness of tadalafil alone or in combination with tamsulosin, for the treatment of BPO.

Previous reviews on this topic have been limited by the inclusion of a small number of studies and insufficient sample sizes [14, 15], potentially leading to inadequate statistical power to detect significant differences in key clinical outcomes. To address these limitations, we conducted an updated meta-analysis to assess the comparative efficacy and safety of combination therapy with tamsulosin and tadalafil versus monotherapy in BPO patients with or without erectile dysfunction.

## Methods

This meta-analysis has been conducted under principles and guidelines outlined in the Cochrane Handbook for Systematic Reviews of Interventions and aligns with the PRISMA (Preferred Reporting Items for Systematic Reviews and Meta-Analyses) reporting standards [16, 17]. No ethical approval was necessary for this study.

### Data sources and searches

We searched Cochrane Library, PUBMED, and Embase from inception until August 2024. A search strategy was formulated using MeSH terms and Emtree terms such as"tamsulosin","LUTS", and"BPH","tadalafil", and"randomized controlled trial”. Boolean operators (AND, OR) were employed in the search terms. Additionally, a partial search of grey literature was conducted via Google Scholar, supplemented by backward citation tracking from the reference lists of pertinent studies. The detailed search strategy is presented in Supplementary Table 1.

### Eligibility criteria

Studies were considered suitable for inclusion in our meta-analysis if they met the following criteria: (1) Study design: randomized controlled trials; (2) Population: Patients with BPH with or without erectile dysfunction; (3) Intervention: tamsulosin plus tadalafil; (4) Comparator: tamsulosin alone; (5) Outcome: reporting at least one outcome of interest.

The exclusion criteria included: (1) Studies with designs other than cohort or randomized controlled trials, such as editorial articles, correspondences, post-hoc analyses, and reviews; (2) Animal studies.

### Study selection

The studies identified through the search strategy were imported into EndNote 21. Following the removal of duplicates, articles were screened based on their titles and abstracts by two authors. Full texts of eligible articles were subsequently retrieved for further screening. Any disagreements between the two authors were resolved by a third senior author.

### Data extraction and outcomes

Data on study characteristics, including author names, study location, sample size, mean age, and dosage were extracted from eligible studies. Furthermore, baseline variables like BMI, overall International Prostate Symptom Score (IPSS), IPSS storage, IPSS voiding, QoL score, maximum urine flow rate (Qmax), postvoid residual volume (PVR), International Index of Erectile Function (IIEF), and adverse events were also extracted. A pre-piloted Excel sheet was utilized to organize this data by two independent authors. The primary outcomes included change in overall IPSS, IIEF, and QoL scores. Secondary outcomes included PVR, Qmax, IPSS storage, IPSS voiding, pain and overall adverse effects.

### Bias assessment

The bias assessment of included randomized controlled trials (RCTs) was conducted with the Risk of Bias tool for randomized studies (RoB 2.0). Three independent reviewers carried out the assessments, and any disagreements were settled by consultation with an additional reviewer.

### Data analysis

Statistical analyses were conducted using R (version 4.4.1) with the'meta'package. Dichotomous outcomes were assessed by calculating Odds Ratios (ORs) with 95% confidence intervals (CIs), while continuous outcomes were evaluated using pooled Weighted Mean Differences (WMDs) with corresponding 95% CIs. To account for heterogeneity among studies, the DerSimonian and Laird random-effects model was applied. To assess the degree of statistical heterogeneity among the included trials, the I^2^ statistic was employed. For outcomes involving more than 10 studies, a meta-regression analysis was conducted using a random-effects model to assess the impact of age and follow-up duration on the pooled estimates. To visually interpret the findings, meta-regression bubble plots were generated. Publication bias was assessed using a funnel plot. Egger’s test and Begg and Mazumdar Rank Correlation were applied to evaluate funnel plot asymmetry. A P-value of less than 0.05 was considered indicative of publication bias. To explore the high heterogeneity, leave-one-out sensitivity analyses were performed, where each study was sequentially removed to determine its influence on aggregate findings. A p-value < 0.05 indicated statistical significance.

## Results

### Study selection

**Fig. 1:**
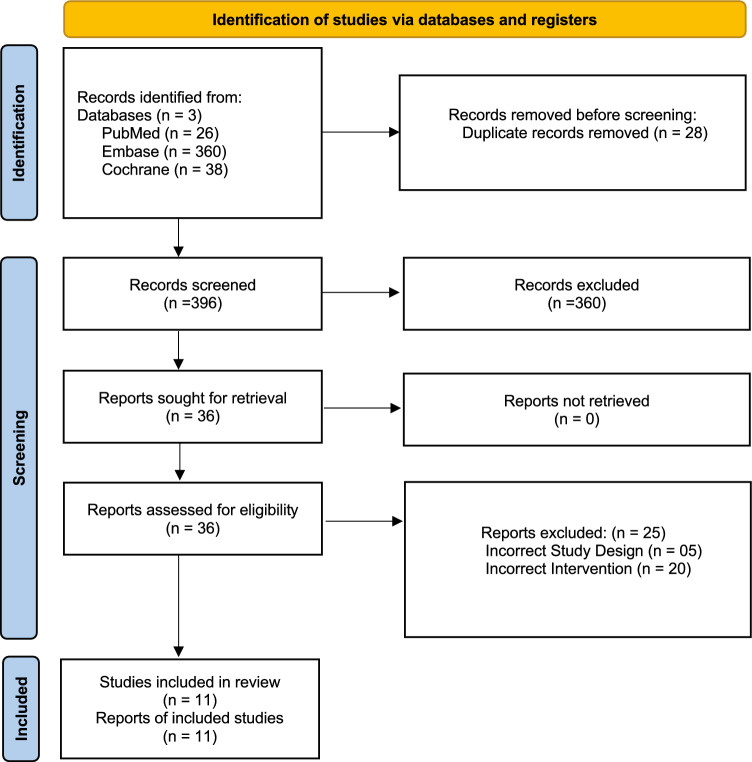
PRISMA flowchart depicting the screening and study selection process

The search yielded 424 articles. After removing 28 duplicate records, 396 records remained for screening based. Out of these, 360 records were excluded, leaving 36 reports selected for full-length screening. Ultimately, 11 studies met the inclusion criteria. (Fig. 1).

### Characteristics of included studies

Our analysis included a total of 940 patients with benign prostatic hyperplasia (BPH), with or without erectile dysfunction, who were treated with either tamsulosin alone or in combination with tadalafil. Tadalafil doses ranged from 5 to 20 mg per day, while tamsulosin was administered at 0.2 or 0.4 mg daily. All included studies were randomized controlled trials. Study characteristics, including sample sizes, mean age, dosing regimens, and study designs, are summarized in Table 1. Detailed baseline characteristics are highlighted in Table 2.

**Table 1:** Characteristics of Included studies

Study ID	Study design	Location	Mean age		Sample size			Dose	
			TAM/TAD	TAM	TAM/TAD	TAM	Total	TAM/TAD	TAM
Singh (2014)	RCT	India	61.92 ± 6.291	59.50 ± 6.048	45	44	89	0.4 mg/day plus 10 MG/day	0.4 mg
Sharma (2016)	RCT	India	45–65	45–65	38	54	92	0.4 mg/day plus 5 mg/day	0.4 mg
Regadas (2013)	RCT	Brazil	61.6 (± 1.7)	59.2 (± 2.0)	20	20	40	0.4 mg/day plus 5 mg/day	0.4 mg
Negoro (2019)	RCT	Japan	73 (65‐85)	70 (50‐80)	13	13	26	− 0.2 mg/day plus 5 mg/day	− (maybe 0.2 MG)
Nasef (2021)	RCT	Egypt	60.51 ± 6.93	62.05 ± 6.87	41	41	82	0.4 mg/day plus 5 mg/day	0.4 mg
Nagasubramanian (2020)	RCT	India	58.87 (8.16)	61.28 (8.23)	69	71	140	0.4 mg/day plus 5 mg/day	0.4 mg
Karami (2016)	RCT	Iran	67.9 ± 8.8	68.5 ± 8.9	58	59	117	0.4 mg/day plus 20 mg/day	0.4 mg
Bechara (2008)	RCT	Argentina	63.7 (51–78)		15	15	30	0.4 mg/day plus 20 mg/day	0.4 mg
Chuanxing (2012)	RCT	China	50. 09 ± 9. 18	51. 27 ± 9. 87	49	48	97	0.2 mg/day plus 10 mg/day	0.2 mg
Dellatti (2013)	RCT	Italy	64.2 ± 10.20	63.8 ± 10.15	49	48	97	0.4 mg/day plus 5 mg/day	0.4 mg
Wang (2020)	RCT	China	34.3 ± 7.6	60.7 ± 7.1	65	65	130	0.2 mg/day plus 5 mg/day	0.2 mg

Continuous data is reported either as mean ± standard deviation or Median/Range IQR

**Table 2:** Baseline characteristics of individual study

Study, year	Age		BMI (kg/m^2^)		QoL		IIEF		PV (mL)		PSA (ng/mL)		Overall IPSS		Qmax (mL/s)	
	TAM + TAD	TAM	TAM + TAD	TAM	TAM + TAD	TAM	TAM + TAD	TAM	TAM + TAD	TAM	TAM + TAD	TAM	TAM + TAD	TAM	TAM + TAD	TAM
Singh (2014)	61.92 ± 6.291	59.50 ± 6.048	NA		5.65 ± 0.562	5.59 ± 0.501	10.61 ± 5.582	10.08 ± 5.064	NA		≤ 4	≤ 4	21.73 ± 5.876	20.93 ± 4.607	9.88 ± 3.581	9.15 ± 3.022
Sharma (2016)	45–65		NA		4.76 ± 0.78	4.81 ± 0.99	NA		NA		NA		24.32 ± 2.68	23.06 ± 2.23	10.82 ± 0.76	10.80 ± 1.25
Regadas (2013)	61.6 ± 1.7	59.2 ± 2.0	NA		NA		NA		44.3 ± 1.8	42.4 ± 2.1	NA		20.6 ± 3.9	20.4 ± 4.3	6.2 ± 2.6	7.4 ± 2.6
Negoro (2019)	73 (65‐85)	70 (50‐80)	23.2 (16.9–28.4)	23.2 (13.7–27.2)	4 (3–5)	5 (3–6)	NA		30.0 (22.0–39.7)	32.0 (20.1–39.5)	NA		17 (10‐27)	16 (10‐24)	8.5 (3.1‐21.2)	12.6 (4.6‐25.5)
Nasef (2021)	60.51 ± 6.93	62.05 ± 6.87	NA		3.37 ± 0.62	3.44 ± 0.63	NA		43.76 ± 6.69	47.71 ± 7.71	2.94 ± 0.63	2.92 ± 0.62	12.15 ± 3.93	12.46 ± 3.71	11.66 ± 1.93	12.29 ± 1.94
Nagasubramanian (2020)	58.87 ± 8.16	61.28 ± 8.23	24.17 ± 3.50	24.39 ± 3.38	4.55 ± 0.85	4.23 ± 0.90	10.06 ± 3.48	9.93 ± 4.08	NA		NA		16.26 ± 3.32	15.10 ± 3.96	9.75 ± 2.17	9.89 ± 3.11
Karami (2016)	67.9 ± 8.8	68.5 ± 8.9	27.1 ± 2.3	26.7 ± 2.4	4.1 ± 1.2	3.9 ± 1.2	10.6 ± 1.7	10.9 ± 1.6	63.2 ± 12.1	61.1 ± 16.1	2.1 ± 1.6	2.3 ± 1.9	21.2 ± 7.5	20.6 ± 7.3	12.4 ± 4.8	12.3 ± 3.8
Bechara (2008)	63.7 (51–78)		NA		4.1 (0–6)	4.1 (0–6)	17 (1–29)	17 (1–29)	NA		≤ 4	≤ 4	19.4 (12–34)	19.4 (12–34)	9.6 (4–14)	9.6 (4–14)
Chuanxing (2021)	50.09 ± 9.18	51.27 ± 9.87	22.67 ± 2.81	22.16 ± 2.96	NA		NA		41.23 ± 9.15	42.48 ± 9.01	NA		25.78 ± 4.38	26.45 ± 4.47	8.13 ± 3.02	7.98 ± 2.87
Dellati (2013)	64.2 ± 10.20	63.8 ± 10.15	NA		2.56 ± 0.88	2.94 ± 0.98	13.4 ± 4.2	11.6 ± 2.9	NA		NA		13.66 ± 4.35	12.87 ± 3.84	9.09 ± 2.91	8.74 ± 2.32
Wang (2020)	61.2 ± 5.9	60.7 ± 7.1	NA		4.7 ± 0.7	4.9 ± 0.8	NA		34.3 ± 7.6	32.1 ± 6.4	NA		17.5 ± 3.8	16.9 ± 4.1	7.8 ± 3.1	7.4 ± 3.3

*BMI* body mass index, *QoL* quality of life, *IIEF* International Index of Erectile Function, *PV* prostate volume, *PSA* prostatic-specific antigen, *IPSS* international prostatic symptoms score, *Qmax* maximum flow rate, *TAM* tamsulosin, *TAD* tadalafil

Continuous data is reported as mean ± standard deviation or Median/Range IQR

### Risk of bias

Six studies had an overall low risk of bias. Five studies had some concerns of bias, primarily due to the conerns in domains of randomization process, measurement of the outcome, and deviations from intended interventions. The details of the bias assessment for each included study are shown in Figure S1.

### Primary outcomes

#### Overall IPSS

**Fig. 2:**
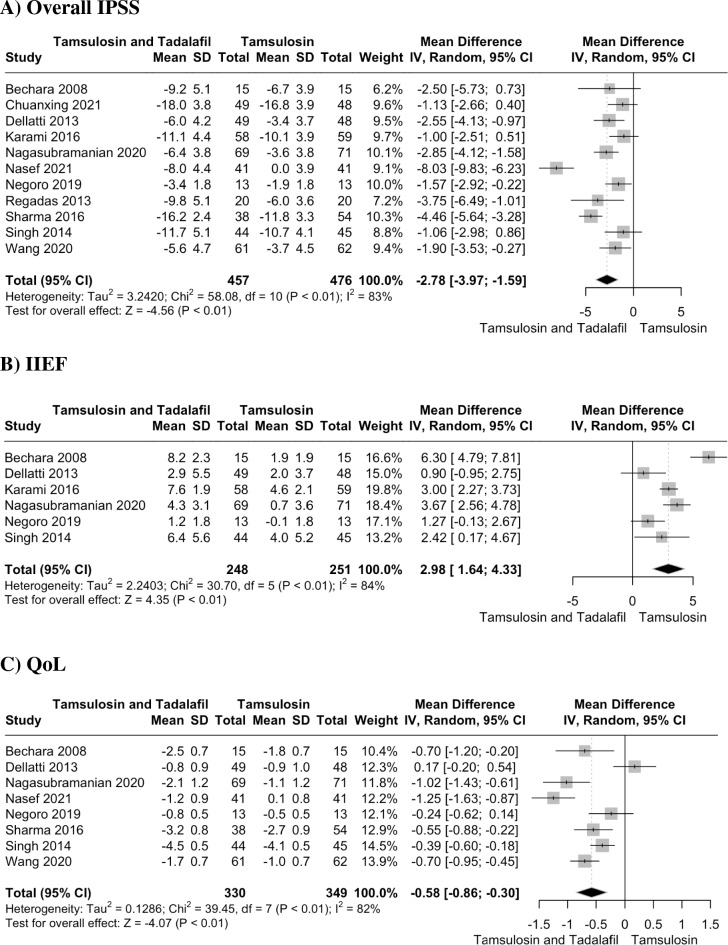
Forest plot showing changes from baseline in **A** Overall IPSS, **B** IIEF, **C** QoL

Eleven studies with 933 patients reported the change in overall IPSS. The pooled analysis showed that the combination group experienced a significantly greater reduction in overall IPSS compared to the control group (MD = − 2.78, 95% CI − 3.97 to − 1.59; P < 0.01) (Fig. 2A). The heterogeneity was substantial (I^2^ = 83%). A leave-one-out analysis by excluding the study by Nasef et al. reduced the I^2^ to 61% (Figure S2). No asymmetry was noted in the funnel plot (Egger’s test, P = 0.9) (Figure S3). Meta regression showed no significant moderating effect of age (estimate = 0.06, 95% CI − 0.17 to 0.28, p = 0.6, R^2^ = 0%) or follow-up duration (estimate = 0.5, 95% CI − 0.99 to 2.0, p = 0.5, R^2^ = 0%) on overall IPSS (Figure S4–S5). IPSS storage and voiding sub-scores have also been analyzed. Only three studies reported on these. The pooled analysis demonstrated significant reduction in IPSS storage (MD = − 0.60, 95% CI − 1.18 to − 0.02; p = 0.04) (Figure S6) and voiding sub-scores (MD = − 1.14, 95% CI = − 1.75 to − 0.52; p < 0.01, I^2^ = 0) (Figure S7) in combination therapy as compared to tamsulosin alone. IPSS storage subscore demonstrated substantial heterogeneity (I^2^ = 69%), which reduced to zero (P < 0.04) on omitting the study by Regadas et al. (Figure S8).

#### IIEF

Six studies reported IIEF with total of 499 patients. The combination group showed a significantly greater improvement in IIEF score compared to the tamsulosin-alone group (MD = 2.98, 95% CI 1.64 to 4.33; P < 0.01) (Fig. 2B). The substantial heterogeneity (I^2^ = 84%) prompted for a leave-one-out analysis. Omitting the study by Bechara et al. lowered heterogeneity to 65% (Figure S9).

#### QoL

Eight studies with a total of 679 patients reported data on change in QoL score. The combination therapy was associated with a significant reduction in QoL score (MD = − 0.58, 95% CI − 0.86 to − 0.30; P < 0.01) (Fig. 2C) compared with tamsulosin alone. The heterogeneity was substantial (I^2^ = 82%). It was determined that heterogeneity was not due to any specific study (Figure S10).

### Secondary outcomes

#### PVR

**Fig. 3:**
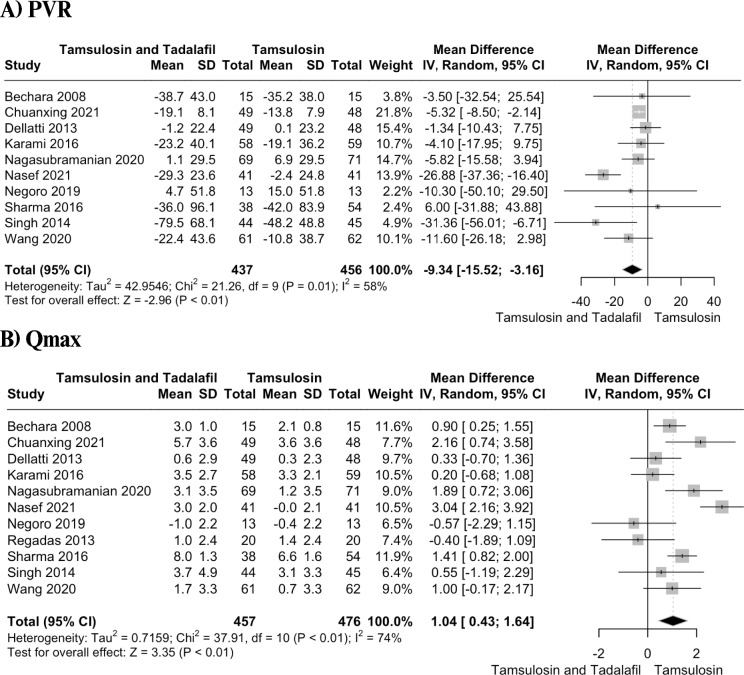
Forest plots depicting change in **A** PVR, **B** Qmax

Ten studies with a total of 893 patients reported data on PVR. The results showed significant decrease in PVR in combination group as compared to control group (MD= −9.34, 95% CI = −15.52 to −3.16; P < 0.01) (Figure 3A). The heterogeneity was high (I^2^=58%). Leave-one-out analysis by omitting the study by Nasef et al. reduced heterogeneity to zero (Figure S11). No asymmetry was noted in the funnel plot (Egger’s test, P = 0.9) (Figure S12). Meta-regression showed no significant moderating effect of age (estimate = − 0.09, 95% CI −1.4 to 1.25, p = 0.9, R^2^ = 0%) or follow-up duration (estimate = 2.8, 95% CI −6.1 to 11.8, p = 0.53, R^2^ = 0%) on PVR (Figures S13, S14).

#### Qmax

Eleven studies with a total of 933 reported on the Qmax. The pooled analysis showed that combination therapy led to a significantly greater improvement in Qmax compared to monotherapy (MD = 1.04, 95% CI 0.43 to 1.64; P < 0.01) (Figure 3B). The heterogeneity was substantial (I^2^=74%). Leave-one-out analysis by omitting the study by Nasef et al. reduced heterogeneity to 50% (Figure S15). There was no evidence of publication bias on the inspection of funnel plot asymmetry (Egger’s test, P = 0.5) (Figure S16). Meta-regression analysis showed a significant moderating effect of age on Qmax (estimate = −0.1, 95% CI −0.22 to −0.001, p = 0.04, R^2^ = 18.78%) (Figure S17). No moderating effect of follow-up duration was found (estimate = −0.17, 95% CI −0.91 to 0.56, p = 0.6, R^2^ = 0%) (Figure S18).

#### Any pain

**Fig. 4:**
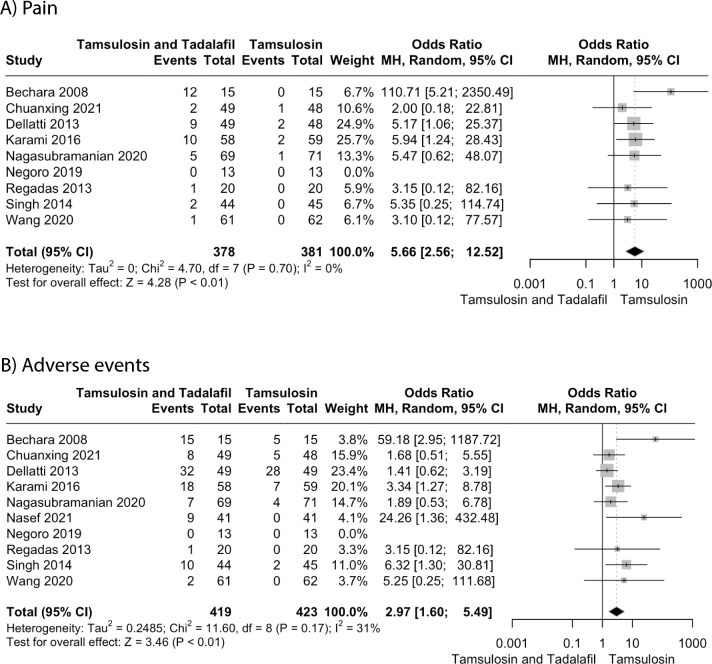
Forest plots for **A** Any pain, **B** Adverse events

Pain included headache, backpain, myalgia, bone pain. Nine studies with a sample of 759 analyzed the severity of pain after taking medicine. The pooled analysis demonstrated that the incidence of pain was significantly higher in combination group as compared to control (OR = 5.66, 95% CI 2.56 to 12.52; P < 0.01) (Fig. 4A). The heterogeneity was minimal (I^2^ = 0%).

#### Overall adverse events

Ten studies with a total of 849 patients evaluated the incidence of AEs. The pooled analysis demonstrated the combination therapy was associated with higher odds of developing adverse effects as compared to monotherapy (OR = 2.97, 95% CI 1.60 to 5.49; P < 0.01, I^2^ = 31%) (Fig. 4B). The heterogeneity reduced to 0% on excluding the study by Bechara et al. (Figure S19).

## Discussion

This review with 11 randomized studies, is an inclusive and thorough analysis of the comparative efficacy of tamsulosin plus tadalafil versus tamsulosin alone in the treatment of benign prostatic obstruction. Our analysis demonstrated that combination therapy is associated with significant improvement in overall IPSS, QoL, PVR, IIEF, and Qmax compared to tamsulosin alone. However, combination therapy resulted in a higher incidence of pain and adverse effects.

Erectile dysfunction (ED) and benign prostatic obstruction (BPO) are conditions closely associated with aging, frequently presenting concurrently in older male patients. This co-occurrence often necessitates a comprehensive approach to management that addresses both lower urinary tract symptoms (LUTS) and sexual dysfunction. Combination pharmacotherapy could emerge as a potentially superior strategy to conventional monotherapy in patients experiencing both conditions.

Our results showed that combination therapy comprising tamsulosin and tadalafil demonstrated significant improvements in LUTS, as evidenced by a marked reduction in the IPSS score. This reduction highlights the efficacy of the combination therapy in alleviating the subjective burden of LUTS compared with tamsulosin monotherapy. The substantial improvement in IPSS underscores the therapeutic advantage of targeting both the dynamic and static components of BPO pathophysiology through dual pharmacological mechanisms. Tamsulosin, an alpha-1 adrenergic receptor antagonist, primarily addresses bladder outlet obstruction by relaxing prostatic smooth muscle, while tadalafil, a phosphodiesterase type 5 (PDE-5) inhibitor, improves LUTS through its effects on smooth muscle relaxation, vascular endothelial function, and prostatic blood flow [10, 18]. Furthermore, the combination therapy yielded significant improvements in the IIEF score, indicating enhanced sexual function among patients concurrently suffering from ED. The observed benefits on the IIEF score suggest that the addition of tadalafil not only complements tamsulosin's action on LUTS but also provides direct therapeutic value for ED. This dual benefit is particularly advantageous in patients for whom ED and LUTS coexist, as it obviates the need for multiple treatment regimens and enhances patient compliance.

The dual approach not only mitigates LUTS but also contributes to improved overall well-being and quality of life. The reduction in IPSS score in combination therapy is complemented by improvements in measures of QoL, suggesting a comprehensive benefit that extends beyond symptomatic relief. These improvements are critical for patients whose daily lives are adversely affected by the interplay of urinary and psychological distress. Our findings align closely with previous meta-analyses; however, we observed a significant difference in IPSS storage subscores and PVR, which were comparable in earlier studies [14, 15]. Additionally, meta-regression analysis revealed a significant moderating effect of age on Qmax, suggesting that older age may attenuate the benefits of combination therapy on Qmax.

Patients with BPO could exhibit severe, life-threatening complications such as acute urinary retention, upper-tract dilation, and renal failure. Consequently, residual urine is a significant focus in efforts to determine the severity of BPO [19]. Historically, the measurement of PVR has been an important test in the evaluation and follow-up of patients with BPO [20]. Nearly all studies included in our analysis reported on Qmax and PVR. These outcomes are highly related to each other as the more the Qmax the lesser the PVR. The combination therapy gave results in favor of both these outcomes being more positively affected. Despite the observed benefits, our analysis revealed a significant increase in the risk of complications and adverse events associated with combination therapy compared to monotherapy. This highlights an important clinical consideration, while combination therapy demonstrates superior efficacy in improving QoL and alleviating symptoms, it also carries a higher burden of potential adverse effects.

Although this review presents an in-depth analysis of the use of tamsulosin plus tadalafil in comparison to tamsulosin alone for the treatment of BPO, it is important to consider the limitations of our review. The quality of these studies is compromised, especially regarding study design, patient selection, blinding, and outcome data, including the potential manipulation or exclusion of data to inflate positive outcomes. Additionally, selection biases and subjective influences must also be accounted for. Studies suggest that an MCID (minimal clinically important differences) of approximately 3 points for IPSS is required for patients to perceive a noticeable improvement in lower urinary tract symptoms (LUTS) [21, 22]. Similarly, for erectile function, a change of 2 to 4 points in IIEF has been associated with a perceptible improvement [23]. Our findings align with these thresholds, suggesting that the observed benefits may have practical significance. However, the improvement in Qmax, while statistically significant, was modest (MD = 1.04 mL/s) and may not lead to a substantial functional difference for all patients [24]. As study-level meta-analysis, the analysis is limited by the lack of access to individual patient data, restricting its ability to consider patient-specific variables and perform more detailed subgroup analyses. To confirm our findings, randomized controlled trials (RCTs) with sufficient sample sizes, accurate data, and extended follow-up periods are necessary.

## Conclusion

The combination therapy of tamsulosin and tadalafil exhibited greater therapeutic efficacy than tamsulosin monotherapy in alleviating lower urinary tract symptoms (LUTS), enhancing quality of life, and improving erectile dysfunction. However, the combination therapy was associated with a higher incidence of adverse effects.

## Supplementary Information

Below is the link to the electronic supplementary material.

Supplementary file1 (DOCX 42054 KB)

## Data Availability

All data generated or analyzed during this study are included in this article. Further inquiries can be directed to the corresponding author. No datasets were generated or analysed during the current study.
