# Supplementary material for: Tamsulosin plus Tadalafil compared with Tamsulosin alone for benign prostate hyperplasia in patients with or without erectile dysfunction: a meta-analysis and meta-regression of randomized controlled trials
Source: World J Urol. 2025 May 11;43(1):291. doi: 10.1007/s00345-025-05662-w (PMC12066372; doi:10.1007/s00345-025-05662-w)

Table S1: Search Strategy

| Database | Search string | Records |
| --- | --- | --- |
| PubMed/MEDLINE | (tamsulosin AND tadalafil) AND (benign prostatic hyperplasia OR BPH OR enlarged prostate) AND ("randomized controlled trial"[Publication Type] OR "randomized controlled trials as topic"[MeSH Terms] OR "RCT"[All Fields]) | 26 |
| Embase | tamsulosin AND tadalafil AND (benign prostatic hyperplasia OR BPH OR enlarged prostate) | 360 |
| Cochrane Library | (tamsulosin AND tadalafil) AND (benign prostatic hyperplasia OR BPH OR enlarged prostate) AND ("randomized controlled trial" OR "RCT") | 38 |

Figure S1: Risk of bias assessment of included studies

**
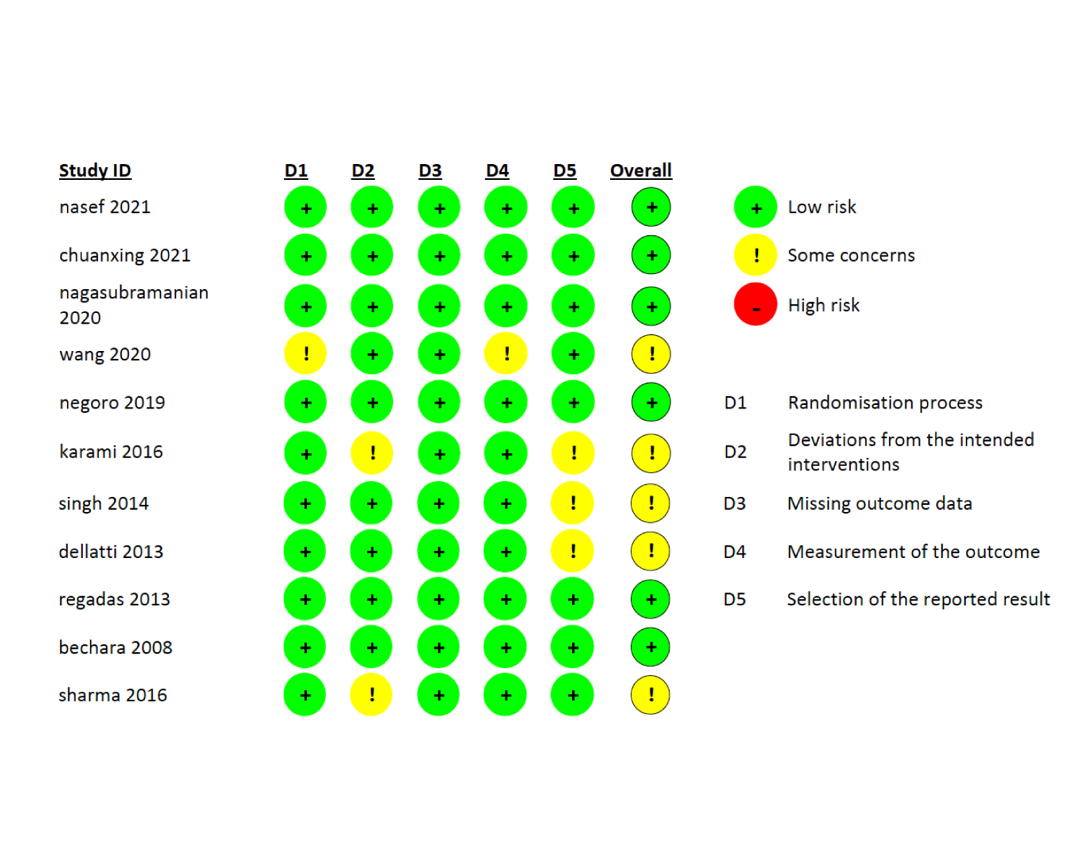
**

Figure S2: Leave-one-out sensitivity analysis for overall IPSS


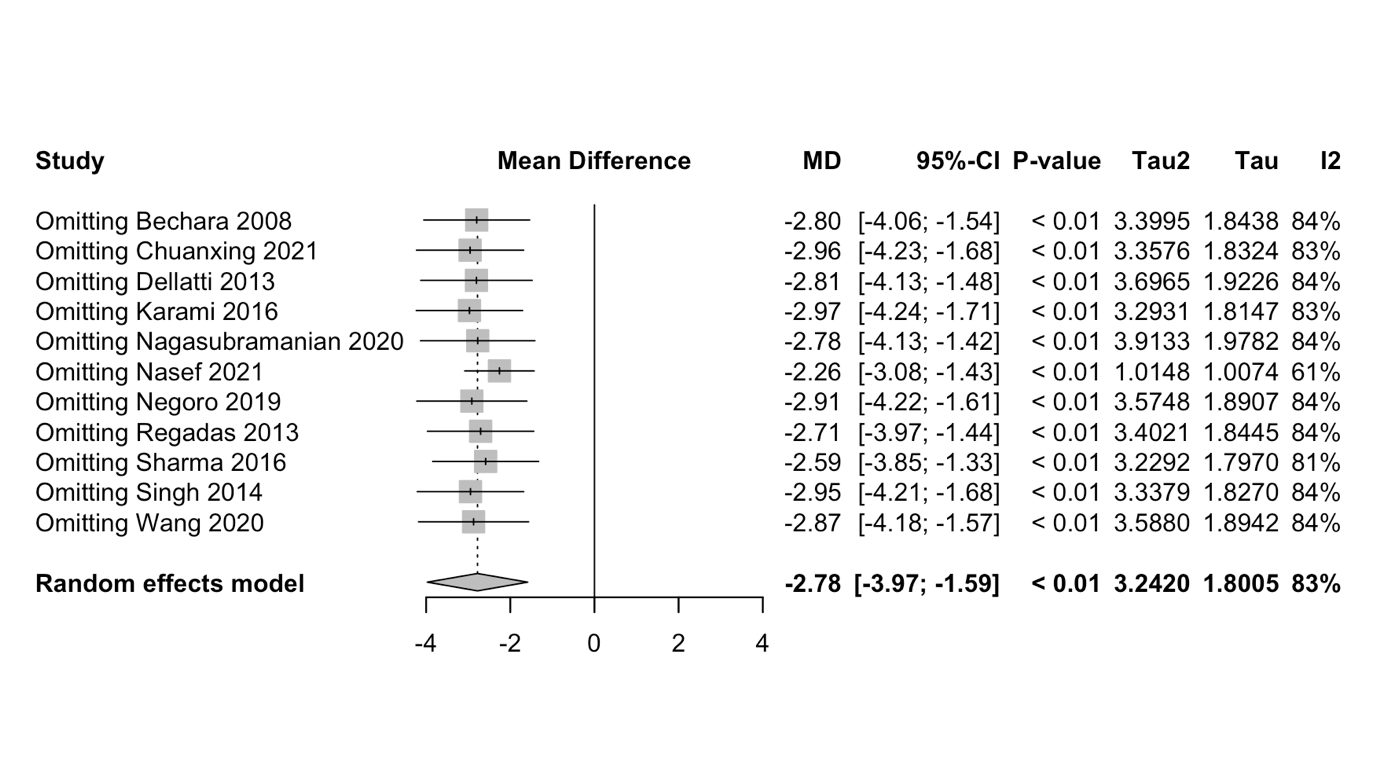


Figure S3: Funnel plot assessing the risk of publication bias for overall IPSS


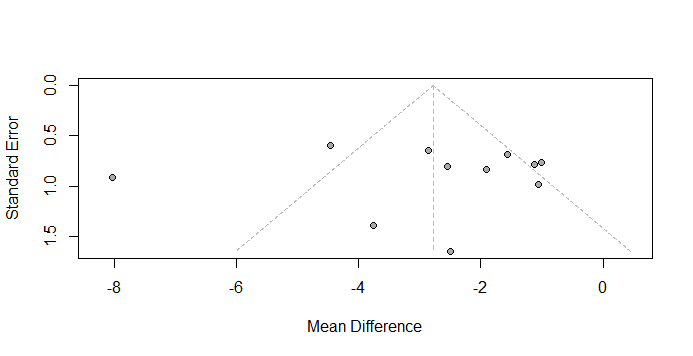


Figure S4: Meta-regression plot showing the effect of age on overall IPSS


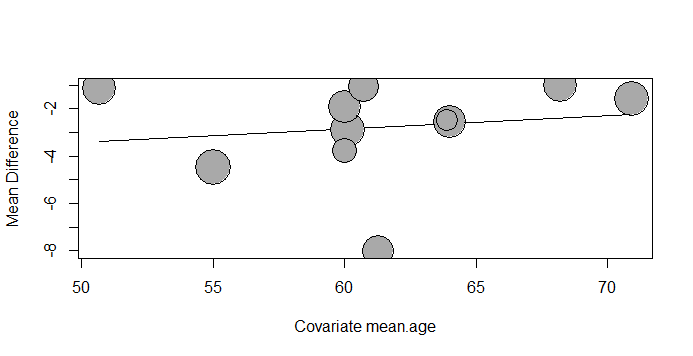


Figure S5: Meta-regression plot showing the effect of follow-up duration on overall IPSS


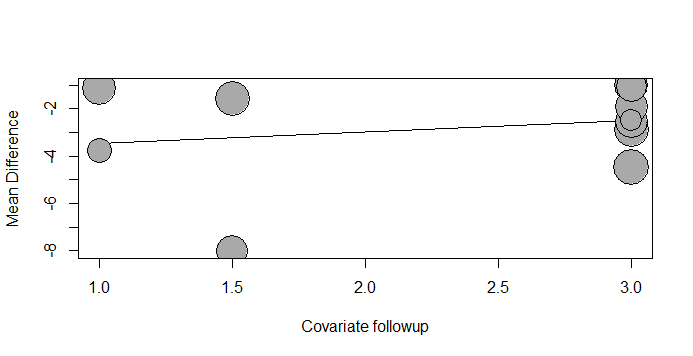


Figure S6: Forest plot for IPSS storage score


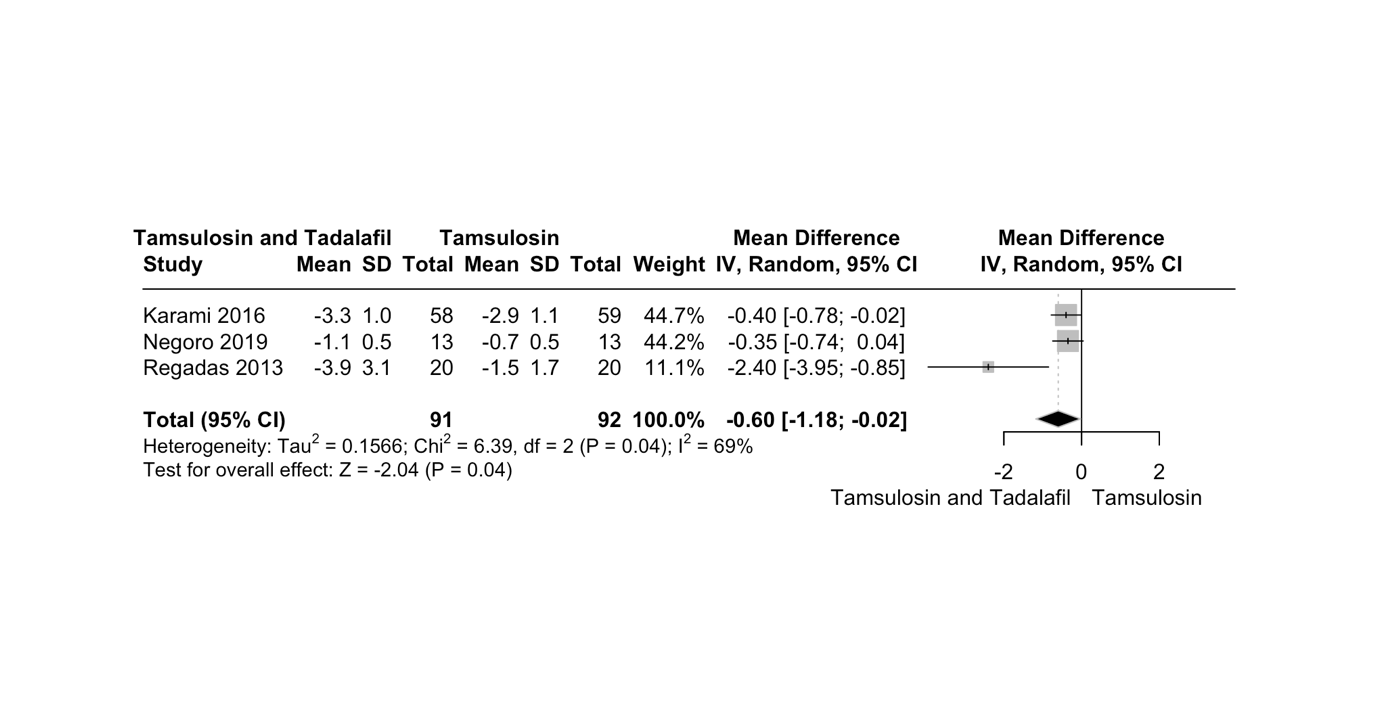


Figure S7: Forest plot for IPSS voiding score


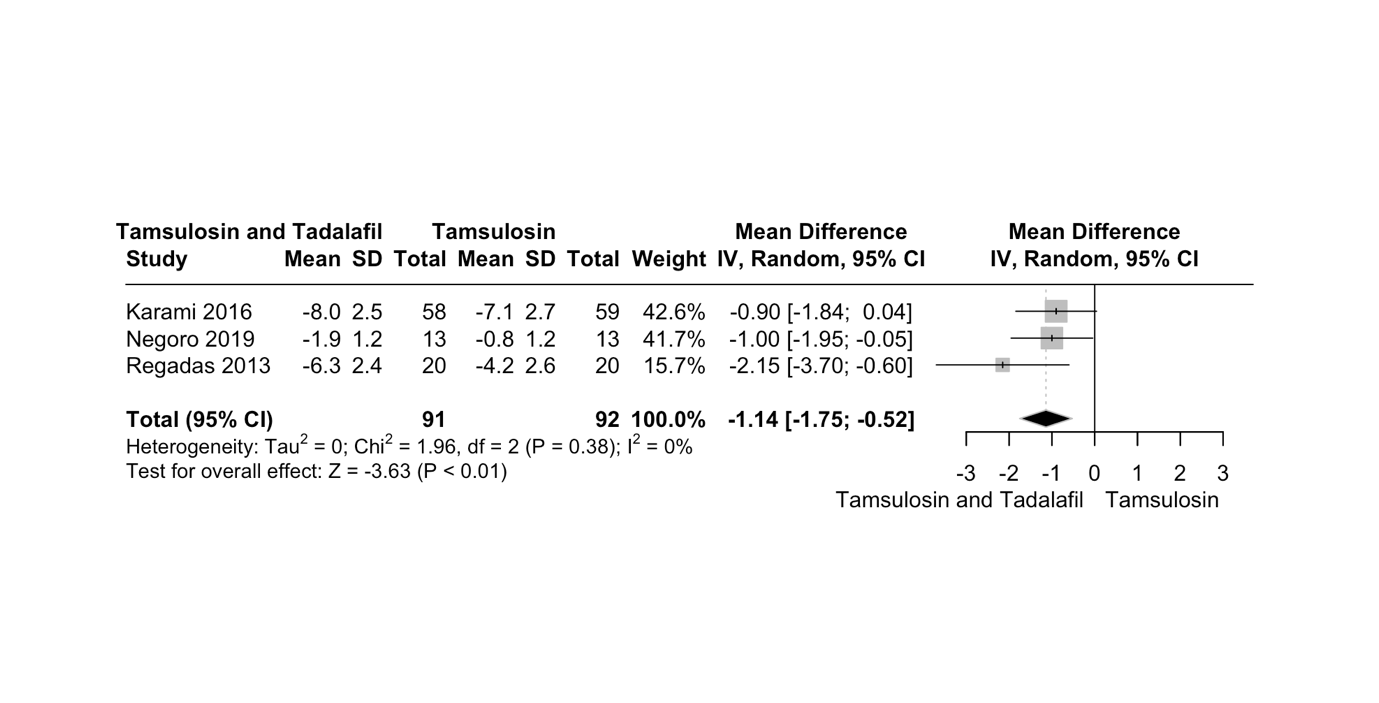


Figure S8: Leave-one-out sensitivity analysis for IPSS storage subscore


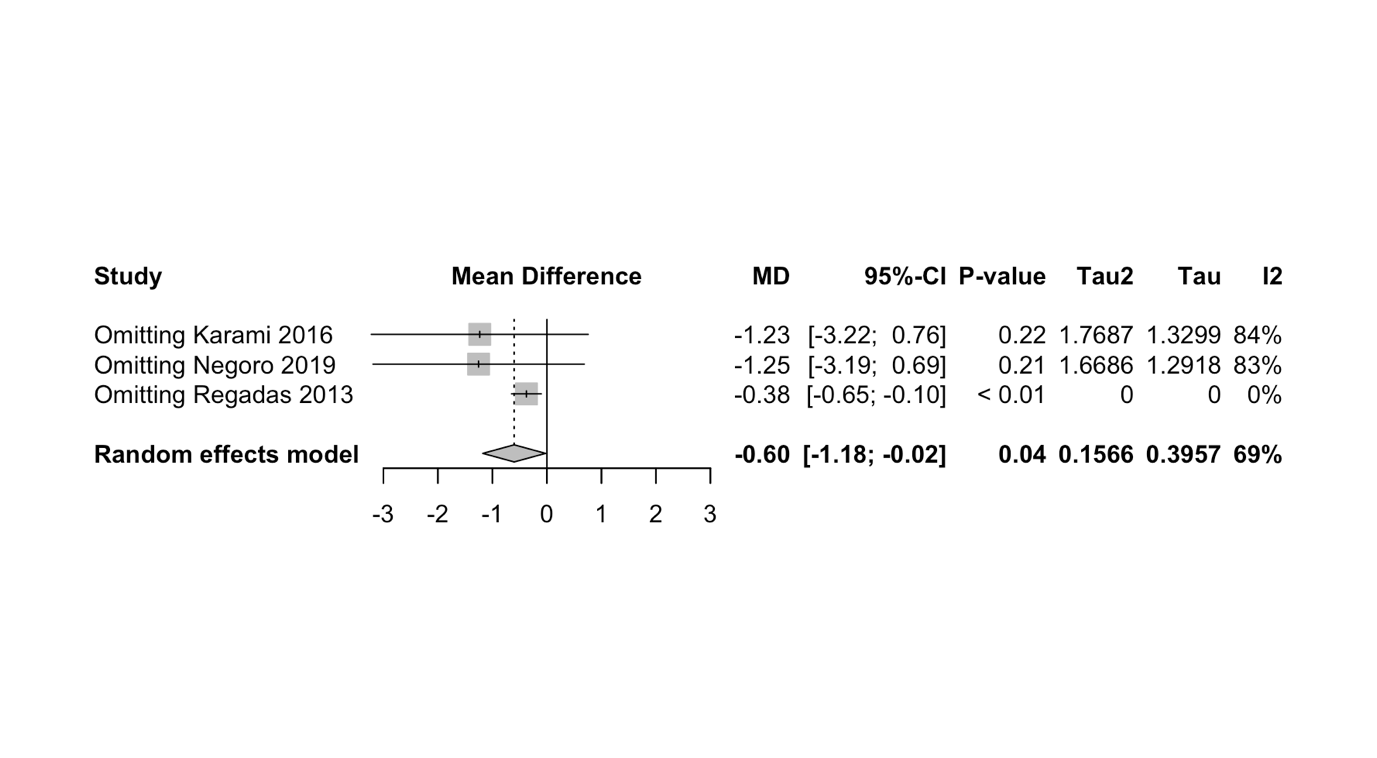


Figure S9: Leave-one-out sensitivity analysis for IIEF


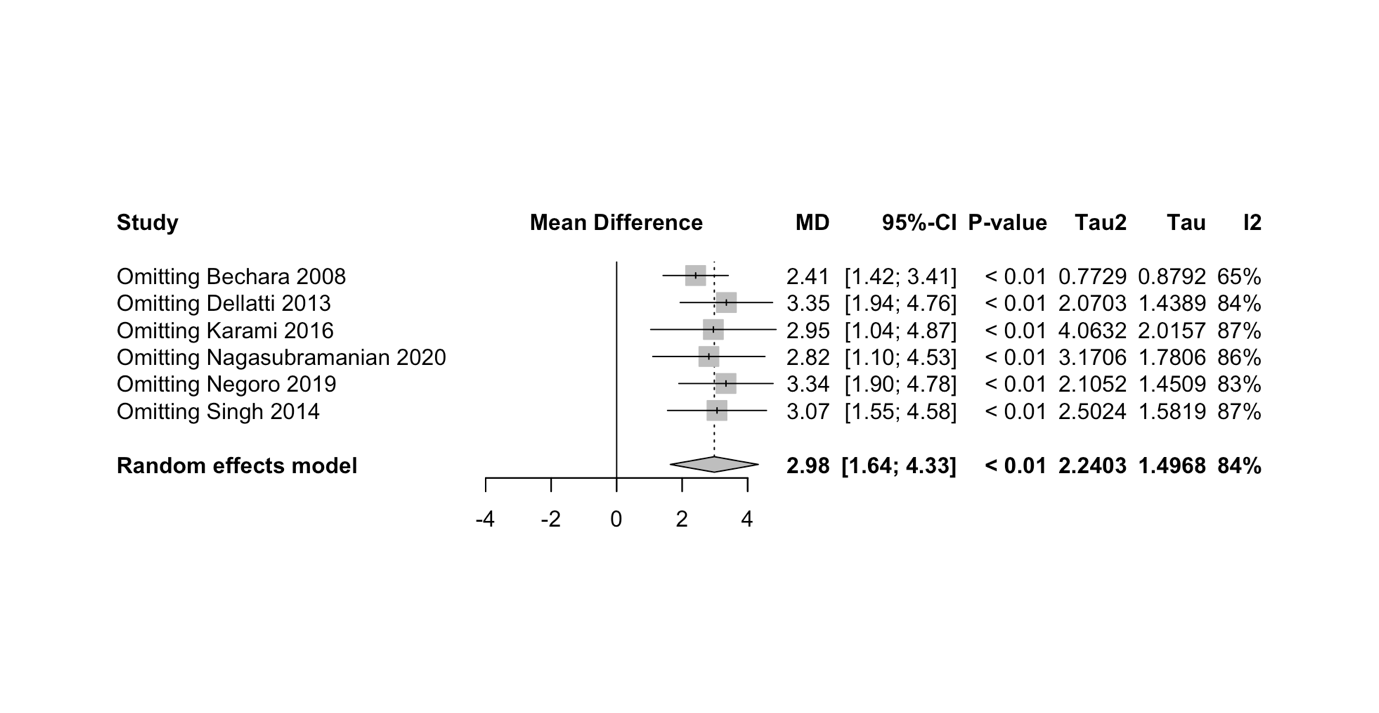


Figure S10: Leave-one-out sensitivity analysis for QoL


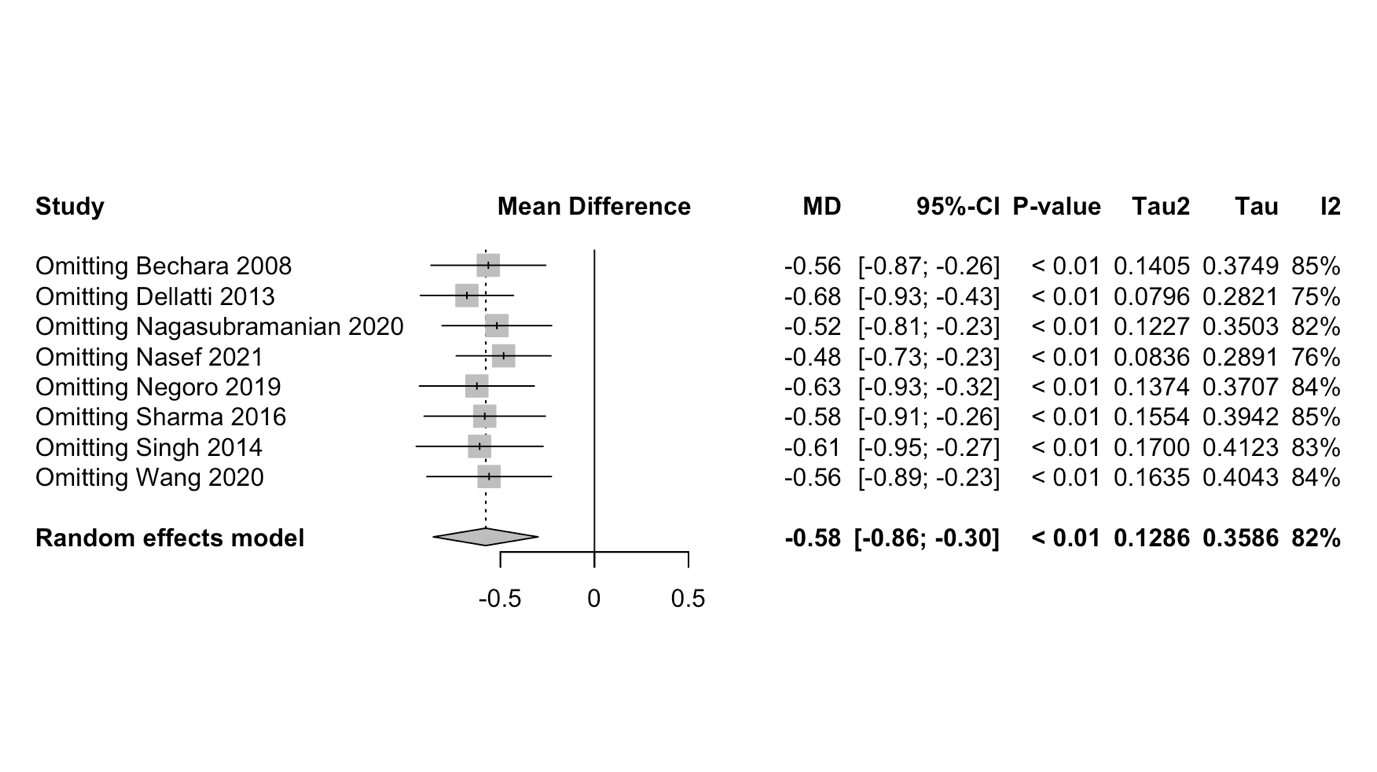


Figure S11: Leave-one-out sensitivity analysis for PRV


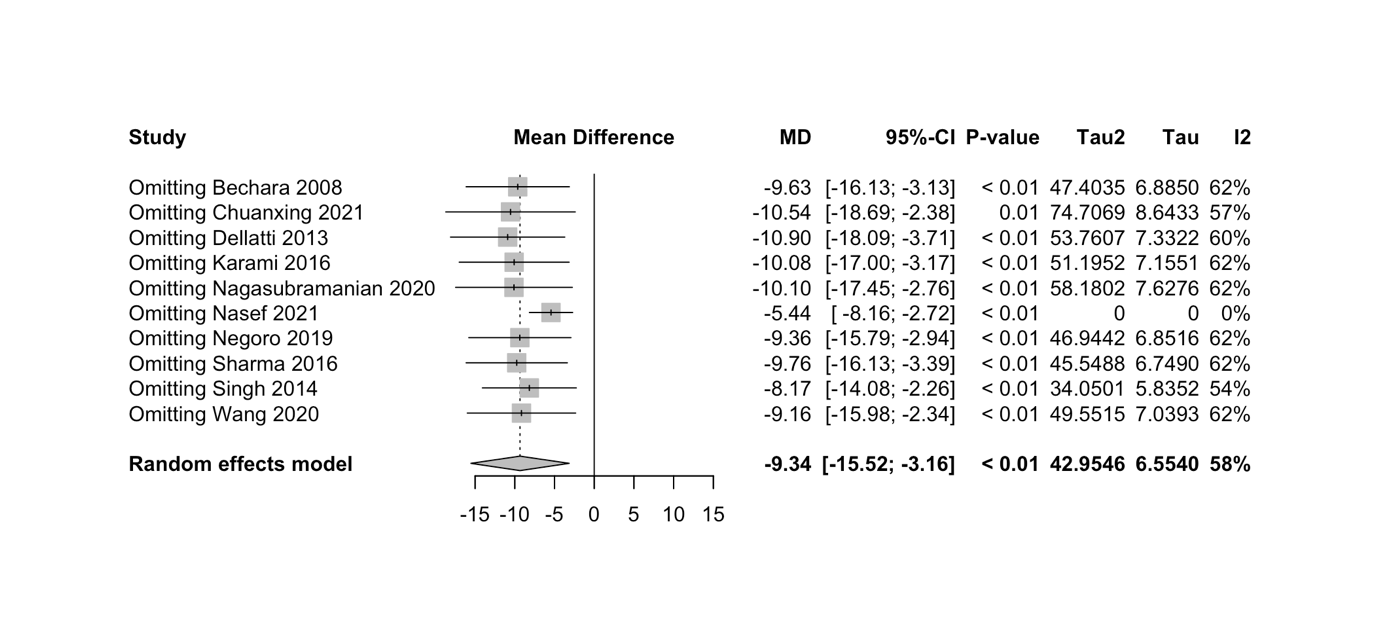


Figure S12: Funnel plot assessing the risk of publication bias for PRV


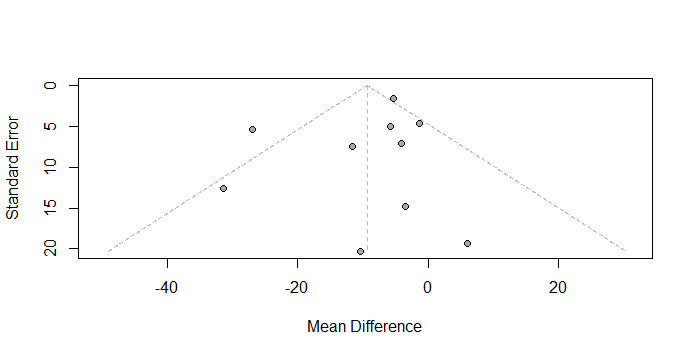


Figure S13: Meta-regression plot showing the effect of age on PRV


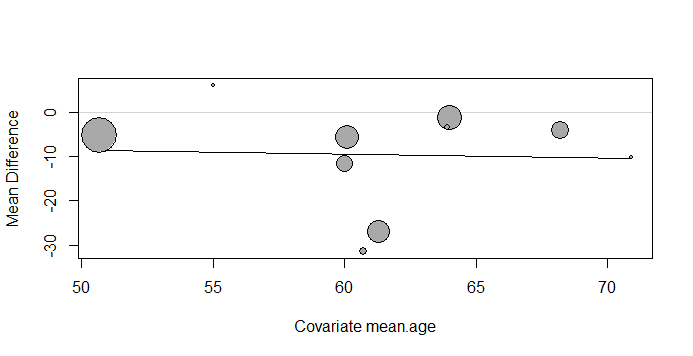


Figure S14: Meta-regression plot showing the effect of follow-up duration on PRV


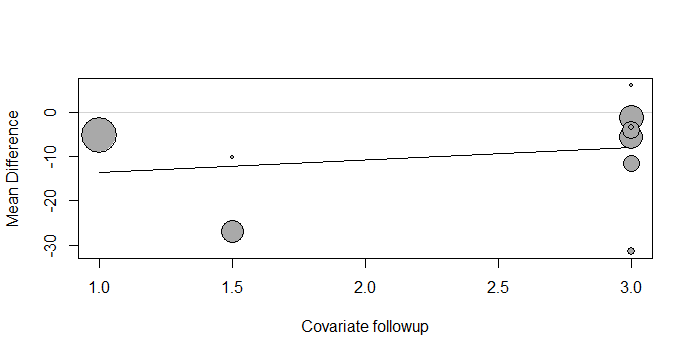


Figure S15: Leave-one-out sensitivity analysis for Qmax


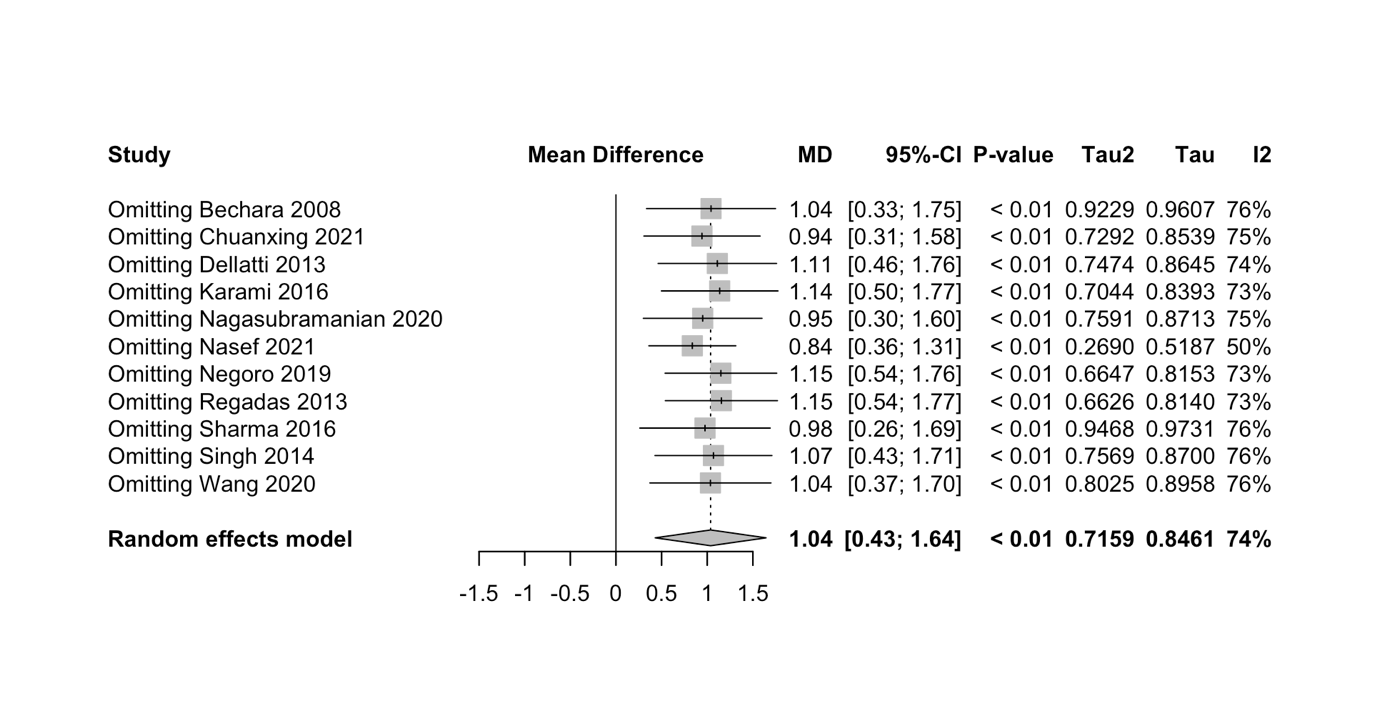


Figure S16: Funnel plot assessing the risk of publication bias for Qmax


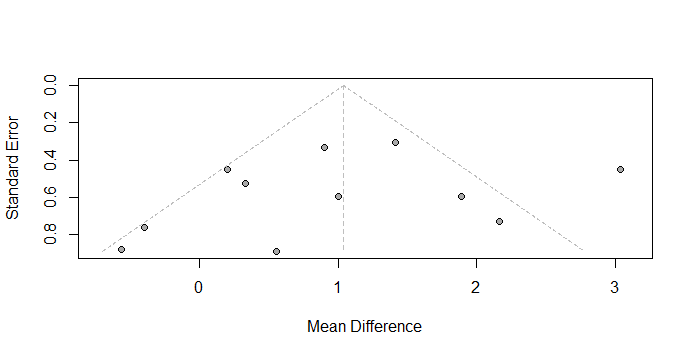


Figure S17: Meta-regression plot showing the effect of age on Qmax


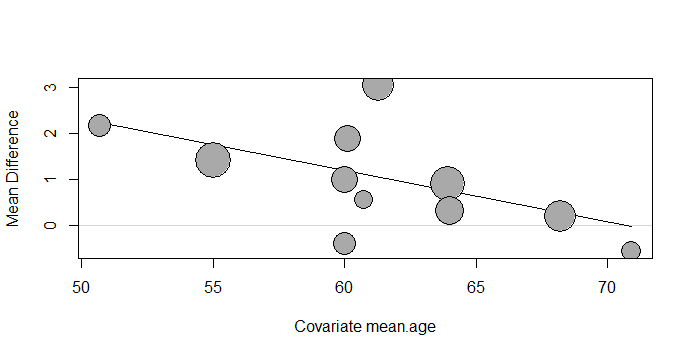


Figure S18: Meta-regression plot showing the effect of follow-up duration on Qmax


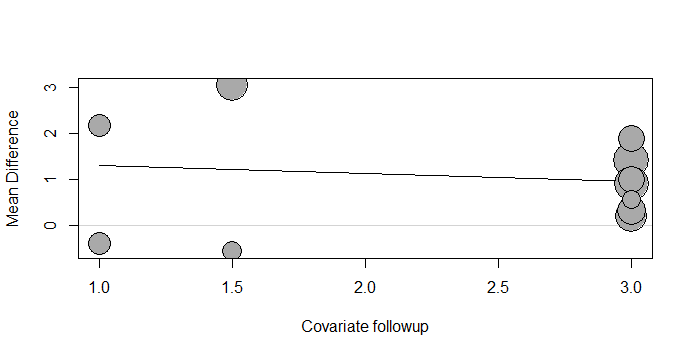


Figure S19: Leave-one-out sensitivity analysis for overall adverse events


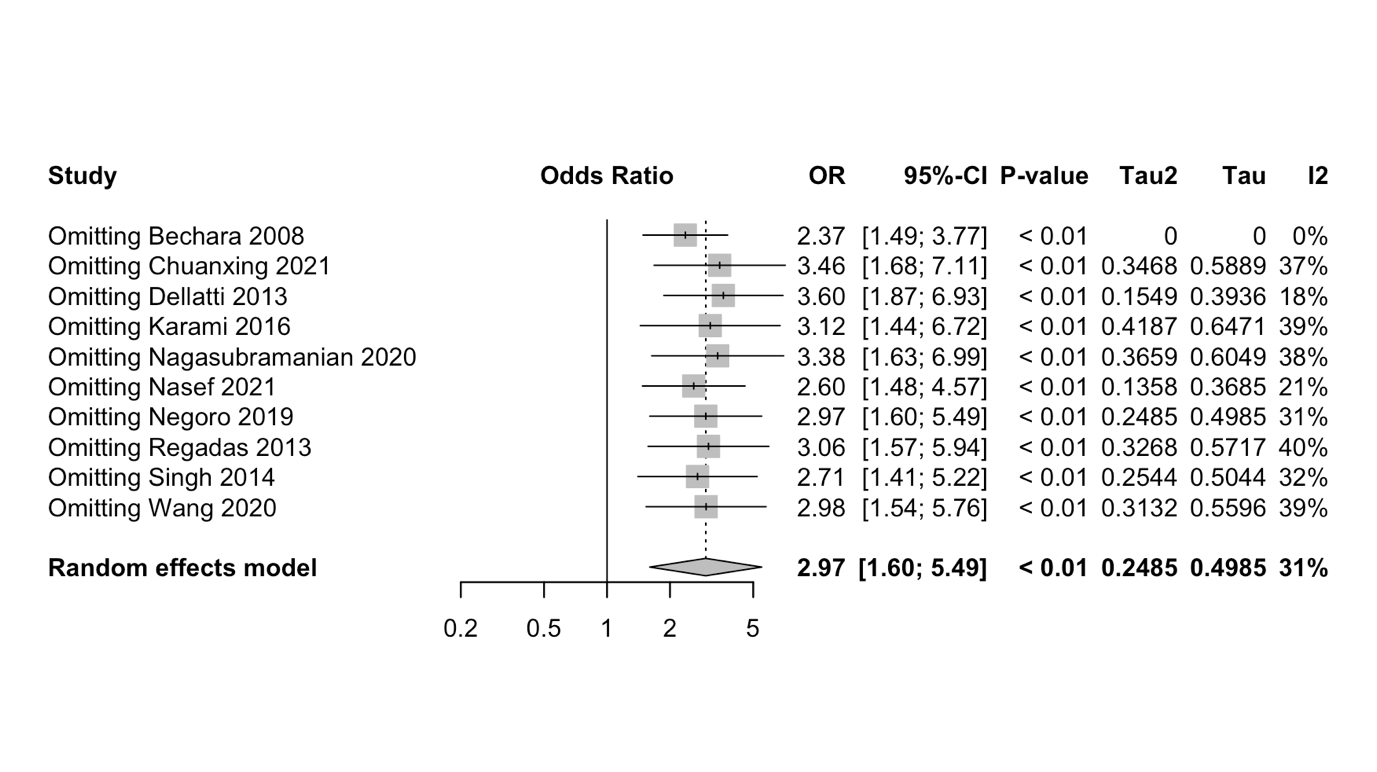

Supplement: Supplementary file 1 — Supplementary file1 (DOCX 42054 KB) [file 345_2025_5662_MOESM1_ESM.docx]
